# Understanding turnover intention among preschool teachers in China: the role of psychological contract violation, organizational cynicism, and equity sensitivity

**DOI:** 10.3389/fpsyg.2026.1734017

**Published:** 2026-03-11

**Authors:** Yibei Yang, Norarbaiyah Binti Yaacob

**Affiliations:** 1 School of Sport, Hainan Normal University, Haikou, Hainan, China; 2 Graduate School of Management, Postgraduate Centre, Management and Science University, Shah Alam, Selangor, Malaysia

**Keywords:** equity sensitivity, organizational cynicism, preschool teacher, psychological contract violation, turnover intention

## Abstract

**Introduction:**

Grounded in social exchange theory, this study examines how psychological contract violation shapes preschool teachers’ turnover intention through cognitive, affective, and behavioral organizational cynicism, and whether equity sensitivity strengthens these links in the Chinese context.

**Methods:**

We surveyed 505 preschool teachers across five Chinese provinces. Using PLS-SEM with 5,000 bootstraps, we tested direct, mediating, and moderating effects among psychological contract violation, three facets of organizational cynicism, equity sensitivity, and turnover intention.

**Results:**

Psychological contract violation shows positive effects on cognitive, affective, and behavioral organizational cynicism as well as on turnover intention. Cognitive organizational cynicism and affective cynicism partially mediate the psychological contract violation-turnover intention relationship, whereas behavioral organizational cynicism does not. Equity sensitivity positively moderates the psychological contract violation-turnover intention path. The model explains 50% of the variance in turnover intention.

**Discussion:**

Findings suggest that distrust and emotional disillusion-rather than overt resistance-translate breached obligations into quitting intentions. Administrators should prioritize promise tracking, procedural justice, and emotional support, with targeted interventions for equity-sensitive staff. Limitations include the cross-sectional design and self-report measures; future work should incorporate longitudinal turnover data.

## 1 Introduction

Psychological contract violation (PCV) is widely regarded as a key antecedent of adverse organizational outcomes, particularly turnover intention (TI) (Sischka et al., 2021; He et al., 2023). Foundational research in organizational psychology has long underscored the centrality of reciprocal exchange relationships in explaining employee withdrawal cognitions and behaviors (Blau, 1964). Currently, in preschool settings, teacher instability is a salient concern, as it erodes team cohesion, degrades instructional quality, and disrupts the continuity of teacher—child interactions. Across early childhood education worldwide, high turnover rates undermine educational quality and institutional stability (Zhang et al., 2024).

In China, these concerns are intensified by the national context. The rapid expansion of preschool education has not been matched by commensurate improvements in compensation, career pathways, and organizational support for teachers, resulting in persistent retention challenges (Liu et al., 2023; Cheng et al., 2025). Recent empirical evidence shows that perceptions of pay equity are negatively related to TI among Chinese preschool teachers, especially those without bianzhi status, and that workload and value perceptions significantly predict retention intention (Liu et al., 2023; Cheng et al., 2025).

Accordingly, this study examines how PCV shapes TI among Chinese preschool teachers. Against this backdrop, the present study investigates the psychological mechanisms through which PCV influences TI among preschool teachers in mainland China, framed within social exchange theory (SET) and complemented by cultural and fairness considerations. Persistent issues in the Chinese early childhood education sector—such as employment insecurity, inequitable compensation, and limited professional development opportunities—underscore the relevance of examining how perceived breaches in implicit psychological contracts contribute to turnover cognitions (Liu et al., 2023).

The effect of PCV on TI is multifaceted, operating through both direct and indirect pathways (Li et al., 2022). However, research on the underlying mechanisms of the PCV—TI linkage in educational contexts remains limited, especially in non-Western settings. To account for this complexity, this study posits OC as a mediator and ES as a moderator. This mediated moderation model applies SET to the Chinese preschool context by integrating ES—a stable individual-difference variable reflecting chronic sensitivity to inequity—as the measured moderator, distinct from general fairness perceptions that serve as the theoretical backdrop for understanding contract breaches (Liu et al., 2023). While fairness perceptions represent situational evaluations of justice, ES captures differential reactivity to inequity.

We conceptualize cultural factors as contextual boundary conditions rather than as separately measured latent variables. In the Chinese context, cultural norms related to collectivism, harmony maintenance, and respect for authority may shape how PCV is construed and how cynical attitudes are expressed, especially limiting overt behavioral cynicism (Wang et al., 2025). Accordingly, we clarify in the theory and discussion how culture may shape each construct and the focal pathways, and we explicitly delimit the cultural scope of inference.

This study contextualizes SET within the Chinese preschool education context by integrating cultural and fairness perspectives into the PCV—TI model, providing critical insights into teacher retention issues in this rapidly expanding sector. Culture is treated as a contextual boundary condition informing theory application rather than an empirically tested extension.

## 2 Literature review

### 2.1 Psychological contract violation and turnover intention

Employees’ unspoken views about duties owed to their company—views not codified in formal contracts—are termed psychological contracts. When workers think their company has not fulfilled its obligations, they experience PCV, which can damage trust, heighten perceptions of unfairness, and undermine the perceived balance in the employment relationship (Li et al., 2022). Meta-analytic evidence confirms that breaches in psychological contract perceptions are associated with negative job attitudes and increased TI across diverse occupational groups (Topa et al., 2022).

In accordance with SET, PCV disrupts the reciprocity expected between employees and organizations. This disruption elicits negative emotions and weakens organizational commitment, thereby elevating TI (He et al., 2023). Empirical studies further indicate that PCV heightens dissatisfaction, disengagement, and emotional exhaustion, which in turn increase TI (Azeem et al., 2020; Zhang et al., 2024).

Despite these established links, much of the existing literature has overlooked the cultural contexts in which PCV occurs. In particular, collectivist cultural norms prevalent in China—such as harmony maintenance and respect for authority—may shape how individuals interpret and respond to perceived contract breaches. This lack of cultural consideration limits the applicability of existing findings to non-Western contexts. This study addresses this gap by examining PCV—TI relationships within the Chinese preschool education context.

### 2.2 Organizational cynicism as a multidimensional mediator

The multifaceted concept of organizational cynicism (OC) is defined by unfavorable attitudes, feelings, and actions directed toward the organization (Pfrombeck et al., 2020). OC encompasses cognitive, affective, and behavioral responses that have been linked to turnover cognition in organizational change and workplace psychology literature (Smith and Jones, 2023).

Cognitive organizational cynicism (OCC) refers to employees’ beliefs that the organization lacks integrity and ethical consistency. Affective organizational cynicism (OCA) reflects emotional reactions such as anger, frustration, and disappointment. Behavioral organizational cynicism (OCB) denotes the outward expression of cynical attitudes.

One key gap in the literature concerns how these dimensions operate in specific cultural contexts. Recent research suggests that cultural norms emphasizing harmony may constrain overt cynical behaviors while still allowing cognitive and affective cynicism to influence TI (Wang et al., 2025). However, much of the existing literature assumes that overt OCB is the primary response to PCV, without considering how collectivist norms may suppress such behaviors. This study highlights how cognitive and affective cynicism, rather than overt behavioral cynicism, may play a more prominent mediating role in predicting TI among Chinese preschool teachers.

### 2.3 Equity sensitivity as a moderator

Equity sensitivity (ES) reflects individual variations in responsiveness to equity or inequity in the work environment (Liu et al., 2023). Employees with high ES tend to respond more strongly to perceived unfairness, exhibiting heightened dissatisfaction and stronger TI.

Although the relationship between ES and TI has been documented, most empirical studies have focused on Western samples (Mensah et al., 2024), with limited attention to collectivist contexts such as China. In collectivist cultures, fairness judgments may be shaped not only by outcome equity but also by relational and normative considerations. This study contributes to the literature by examining how ES moderates the PCV—TI relationship in the Chinese preschool education context.

### 2.4 Theoretical underpinning and hypothesis development

This study is guided by SET, which explains how perceived imbalances in reciprocal exchange relationships influence employee attitudes and behaviors (Blau, 1964). While SET has been widely applied to explain PCV and its outcomes, prior applications have predominantly focused on Western organizational contexts.

By incorporating collectivist cultural norms—such as harmony maintenance and respect for authority—as a contextual backdrop—this study applies SET to examine how PCV, OC, and ES jointly shape TI in China. Cultural factors are treated as contextual boundary conditions rather than as separately measured constructs.

Grounded in this framework, we formulate the following hypotheses:

*H1*: Psychological contract violation is positively associated with turnover intention among preschool teachers in China.

*H2a*: Psychological contract violation is positively associated with cognitive organizational cynicism.

*H2b*: Psychological contract violation is positively associated with affective organizational cynicism.

*H2c*: Psychological contract violation is positively associated with behavioral organizational cynicism.

*H3a*: Cognitive organizational cynicism is positively associated with turnover intention.

*H3b*: Affective organizational cynicism is positively associated with turnover intention.

*H3c*: Behavioral organizational cynicism is positively associated with turnover intention.

*H4a*: Cognitive organizational cynicism mediates the relationship between psychological contract violation and turnover intention.

*H4b*: Affective organizational cynicism mediates the relationship between psychological contract violation and turnover intention.

*H4c*: Behavioral organizational cynicism mediates the relationship between psychological contract violation and turnover intention.

*H5*: Equity sensitivity moderates the relationship between psychological contract violation and turnover intention, such that the relationship is stronger for individuals with high equity sensitivity.

**FIGURE 1:**
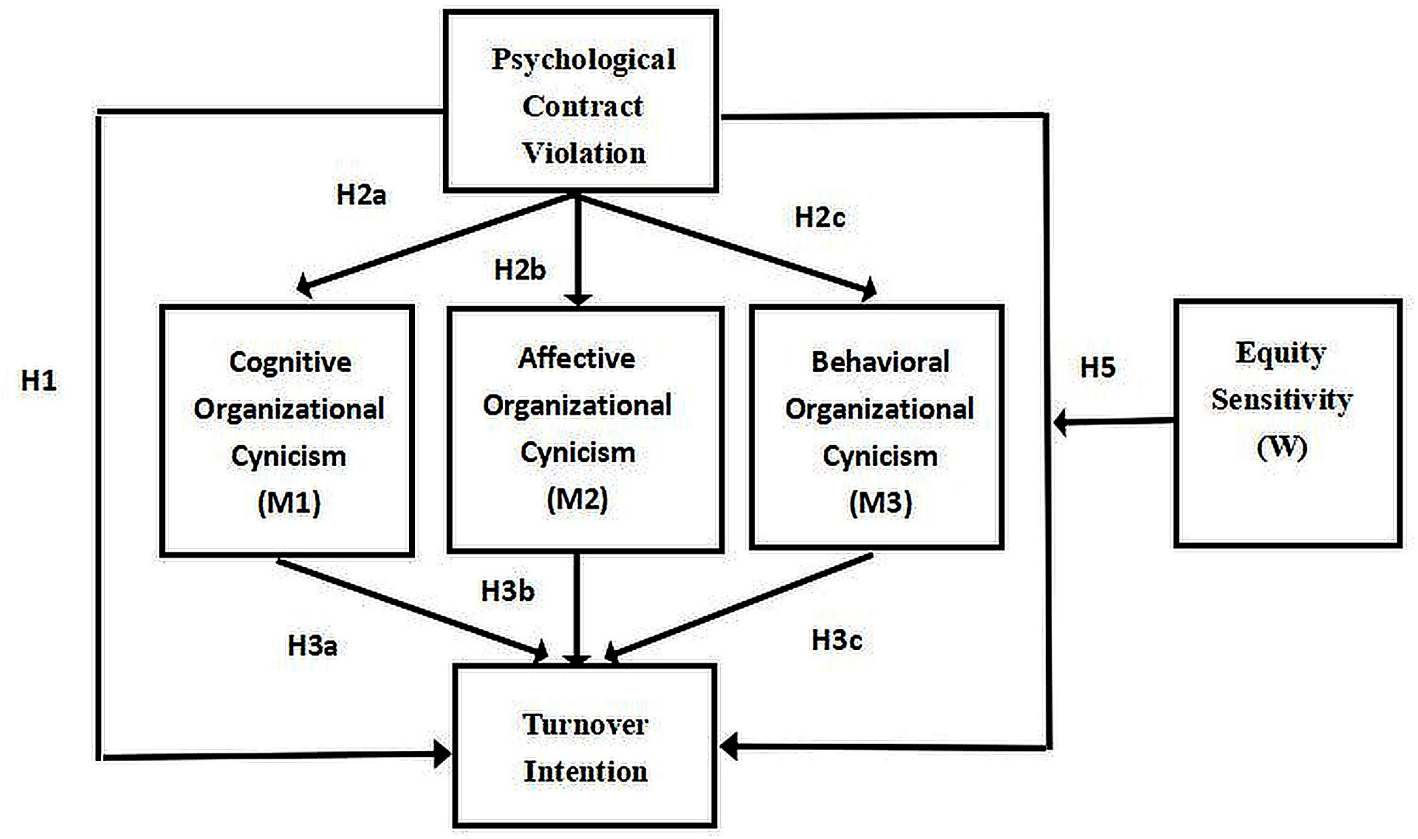
Conceptual framework of the study. H4a–H4c denote the indirect effects of psychological contract violation (PCV) on turnover intention (TI) via cognitive (OCC), affective (OCA), and behavioral (OCB) organizational cynicism, respectively. Equity sensitivity (ES) moderates the PCV—TI relationship (H5).

Figure 1 illustrates the study framework, which is derived from the hypotheses outlined earlier.

## 3 Materials and methods

### 3.1 Research design and procedure

To test theoretical propositions using empirical data, this study adopts a quantitative cross-sectional survey design, in line with Bryman and Bell (2007). We determined the minimal sample size required to attain adequate statistical power (Cohen, 1992). Because the sampling relied on multi-stage non-probability and convenience procedures, statistical representativeness cannot be claimed; instead, we prioritized broad geographic coverage to enhance heterogeneity. Based on an estimated population of 2.83 million preschool teachers in China (Ministry of Education, 2024), the minimum recommended sample size was 384. Sample size: the planned minimum was *n* = 384; the final valid sample was *n* = 505, exceeding the recommended threshold.

Data were collected via Wenjuanxing (an online survey platform). The study was approved by the Research Ethics Committee of Hainan Normal University, in accordance with ethical standards for research involving human participants. All participants provided informed consent before participation, ensuring that their responses would remain confidential and used solely for academic purposes. To facilitate respondent comprehension, the questionnaire was organized into construct blocks aligned with the conceptual model (PCV→OC→TI), and ES was assessed as an individual-difference variable relevant to the focal interaction. This ordering was adopted for survey flow and clarity rather than to impose temporal precedence; given the single-wave design, causal direction is evaluated through theory and robustness checks reported in the Results.

### 3.2 Participants and sampling

To capture regional diversity, we distributed 550 online questionnaires to kindergartens in five strategically selected provinces representing China’s major regions: Jiangsu (East), Guangdong (South), Sichuan (West), Heilongjiang (North), and Henan (Central).

We employed a multi-stage non-probability approach—purposively selecting provinces to stratify by region and, within each province, inviting preschool teachers via participating kindergartens using convenience sampling. Although a non-probability sampling method was employed to ensure broad geographic representation across five provinces, it is important to note that the findings may not be statistically generalizable to the entire population of preschool teachers in China. The non-probability design limits the extent to which the sample can be considered representative of the national population, and thus the results should be interpreted as descriptive of the participating kindergartens rather than generalizable.

We obtained 505 valid responses, yielding a 91.8% valid response rate.

### 3.3 Measurement instruments

Each construct was assessed using internationally established and previously validated scales. To ensure linguistic and cultural equivalence, all instruments underwent professional translation and back-translation between English and Chinese. Demographic variables included gender, age, education level, and income. Although originally validated in general employee samples across organizations, these scales capture non-industry-specific constructs and are therefore applicable to teachers in the education sector. Reliability and validity evidence for the present sample (i.e., measurement model assessment) is reported in Section 4.3.

#### 3.3.1 Psychological contract violation

PCV was measured using the scale originally developed by Robinson and Morrison (2000). In line with prior conceptualizations, PCV was operationalized as employees’ affective response to perceived unmet obligations (violation) rather than a purely cognitive evaluation of contractual breach. A sample item is: “I feel betrayed by my organization.” Responses were recorded on a 5-point Likert scale ranging from 1 = “strongly disagree” to 5 = “strongly agree.” Higher scores indicate a higher level of perceived PCV. Prior research has reported acceptable internal consistency and model fit for this PCV measure (Cronbach’s α = 0.96; χ^2^/df = 1.91, RMSEA = 0.04, CFI = 0.95, TLI = 0.95) (Krivacek et al., 2025).

#### 3.3.2 Organizational cynicism

OC was assessed using Dean et al.’s (1998) 13-item scale, which captures three dimensions: cognitive cynicism, affective cynicism, and behavioral cynicism. The scale consists of five items measuring cognitive cynicism, four items measuring affective cynicism, and four items measuring behavioral cynicism. A sample item for cognitive cynicism is: “I believe that my organization says one thing and does another.” Responses were recorded on a 5-point Likert scale ranging from 1 = “strongly disagree” to 5 = “strongly agree.” Higher scores indicate higher levels of OC. Wu et al. (2025) reported Cronbach’s alpha coefficients of 0.916 (cognitive), 0.905 (affective), and 0.866 (behavioral), with acceptable model fit (χ^2^/df = 1.583, RMSEA = 0.041, CFI = 0.971, TLI = 0.967).

#### 3.3.3 Equity sensitivity

ES was measured using the Equity Preference Questionnaire (EPQ), developed by Sauley and Bedeian (2000), which assesses individual differences in sensitivity to fairness in the workplace. The scale comprises 16 items. A sample item is: “I feel obligated to do more than I am paid to do at work.” Responses were recorded on a 5-point Likert scale (1 = “strongly disagree” to 5 = “strongly agree”). A higher score indicates greater ES. Prior research has reported adequate internal consistency for the EPQ (Cronbach’s α = 0.86) and acceptable CFA fit (χ^2^/df = 6.51, RMSEA = 0.06, CFI = 0.90, TLI = 0.88), supporting its construct validity (Condrea et al., 2021).

#### 3.3.4 Turnover intention

TI was measured using the three-item MOAQ Intent to Quit Scale (Cammann et al., 1983), rated on a seven-point Likert scale. The scale comprises three items. A sample item includes: “I often think about quitting.” Responses were recorded on a 7-point Likert scale ranging from 1 = “strongly disagree” to 7 = “strongly agree.” A higher total score indicates a higher level of TI. Prior research has reported good reliability and excellent model fit for this measure (Cronbach’s α = 0.822; χ^2^/df = 1.137, RMSEA = 0.037, CFI = 0.999, TLI = 0.995) (Chen et al., 2023).

### 3.4 Data analysis strategy

This study employed a combination of SPSS 22.0 and SmartPLS 4.0 for data analysis. Initially, SPSS 22.0 was used to conduct preliminary analyses, including tests for common method bias and descriptive statistics to summarize the demographic characteristics of the sample. Common method variance was assessed using Harman’s single-factor test, and descriptive statistics were calculated to provide an overview of participants’ gender, age, educational background, and income levels.

Subsequently, partial least squares structural equation modeling (PLS-SEM) was conducted using SmartPLS 4.0. PLS-SEM was selected due to its suitability for prediction-oriented research and its robustness in handling complex models involving mediation and moderation effects. Following recommended reporting and inference practices, the statistical significance of model estimates was assessed using non-parametric bootstrapping with 5,000 resamples (Hair et al., 2019). All hypothesis tests were conducted using two-tailed tests with a significance level of α = 0.05. Measurement model evaluation and structural model assessment followed the two-stage procedure recommended by Anderson and Gerbing (1988).

Given the cross-sectional nature of the data, additional analyses were conducted to address potential concerns regarding the assumed ordering between PCV and OC. Specifically, an alternative (reverse) structural model was estimated in which the three facets of OC (cognitive, affective, and behavioral cynicism) were specified as predictors of PCV, while retaining the original paths to TI and the interaction term between ES and PCV. The original and reverse models were compared using both in-sample explanatory power (*R*^2^) and out-of-sample predictive performance indicators for TI, including Q^2^ predict and prediction error metrics (RMSE and MAE), based on PLS predict outputs. These analyses were conducted as robustness checks to examine the stability of the findings rather than to provide definitive evidence of causal direction.

## 4 Results

### 4.1 Common method bias test

To address potential common method bias, we conducted Harman’s single-factor test for common method variance. The results indicated that the first principal factor accounted for 33.1% of the total variance, which is below the 40% threshold commonly used as a cut-off. Therefore, we conclude that common method bias is not a concern in this study.

### 4.2 Descriptive statistics

The survey gathered 505 valid responses and examined participants’ demographic characteristics, including gender, age, educational background, monthly earnings, and additional variables (for more information, refer to Table 1).

**TABLE 1:** Descriptive statistics of the sample.

Construct	Item	N	%
Gender	Male	73	14.5
	Female	432	85.6
Age	< 20	22	4.4
	21–30	217	43
	31–40	169	33.5
	41–50	78	15.4
	> 50	19	3.8
Education	Secondary vocational school degree	11	2.2
	College degree	79	15.6
	Undergraduate degree	338	66.9
	Master’s degree or higher	77	15.2
Income	<2,000	12	2.4
	2,000–3,000	47	9.3
	3,001–4,000	148	29.3
	4,001–5,000	205	40.6
	>5,001	93	18.4

The gender distribution was skewed toward female respondents (85.6%), reflecting the female-dominated nature of the preschool education sector in China. Age: 43% of respondents were aged 21—30, followed by 33.5% aged 31—40. Educational attainment: 66.9% possessed a bachelor’s degree. Income: 40.6% reported monthly incomes of 4,001—5,000 yuan, indicating a moderate income level for the sample. These distributions indicate broad coverage and heterogeneity across key demographics.

### 4.3 Measurement model results

The research hypotheses were evaluated through partial least squares structural equation modeling (PLS-SEM), a commonly employed analytical method known for its reliability (Peng and Lai, 2012). We employed PLS-SEM for two reasons: (a) the primary goal was to predict dependent variables (Roldán and Sánchez-Franco, 2012); and (b) the study’s incremental focus—modeling OC as a mediator between PCV and TI—fits PLS-SEM’s strengths.

In accordance with Anderson and Gerbing (1988), the analysis was conducted in two phases. Initially, we examined the measurement model—evaluating convergent validity, discriminant validity, and internal consistency reliability; subsequently, we assessed the structural model to test the hypotheses. Data were analyzed using SmartPLS 4.0.

#### 4.3.1 Reliability and convergent validity

The measurement model was examined for both reliability and validity. Reliability and convergent validity were evaluated using four key indicators: outer loadings (OL), Cronbach’s alpha (CA), composite reliability (CR), and average variance extracted (AVE). In accordance with established standards, CA (Garver and Mentzer, 1999), CR (Bagozzi and Yi, 1988), and OL (Hulland, 1999) should be ≥ 0.70, while AVE should be ≥ 0.50 for each construct (Chin, 1998; Fornell and Larcker, 1981).

As indicated in Table 2, all outer loadings (OLs) surpassed the 0.70 threshold, ranging from 0.707 to 0.868. Cronbach’s alpha (CA) varied between 0.800 and 0.953, well above the 0.70 cutoff. Composite reliability (CR) ranged from 0.882 to 0.958, exceeding the recommended minimum of 0.70. Average variance extracted (AVE) values ranged from 0.589 to 0.715, all exceeding the 0.50 benchmark. In addition, Table 2 reports the indicator collinearity statistics (outer/indicator VIF). All indicator VIF values ranged from 1.539 to 2.694, well below the recommended cutoffs (3.3 or 5), suggesting that collinearity among indicators is not a concern and that the measurement model estimates are unlikely to be distorted by multicollinearity.

**TABLE 2:** Reliability and validity of the model.

Item	OL	VIF	CA	CR	AVE
ES1	0.806	2.594	0.953	0.958	0.589
ES2	0.788	1.920			
ES3	0.761	2.080			
ES4	0.776	2.043			
ES5	0.759	2.202			
ES6	0.807	2.460			
ES7	0.763	2.090			
ES8	0.821	2.072			
ES9	0.707	2.084			
ES10	0.742	2.470			
ES11	0.731	2.203			
ES12	0.767	2.216			
ES13	0.806	2.155			
ES14	0.745	2.609			
ES15	0.740	2.252			
ES16	0.749	2.694			
OCA1	0.836	1.911	0.821	0.882	0.651
OCA2	0.806	1.619			
OCA3	0.744	1.544			
OCA4	0.838	1.938			
OCB1	0.856	2.144	0.850	0.899	0.689
OCB2	0.829	1.824			
OCB3	0.807	1.807			
OCB4	0.829	1.943			
OCC1	0.838	2.281	0.867	0.904	0.654
OCC2	0.793	1.855			
OCC3	0.763	1.639			
OCC4	0.815	1.963			
OCC5	0.833	2.113			
PCV1	0.814	1.837	0.838	0.892	0.673
PCV2	0.812	1.769			
PCV3	0.832	1.855			
PCV4	0.823	1.837			
TI1	0.868	1.845	0.800	0.882	0.715
TI2	0.804	1.539			
TI3	0.863	1.890			

OL, Outer loadings; CR, composite reliability; AVE, average variance extracted; VIF, variance inflation factor.

#### 4.3.2 Discriminant validity

Discriminant validity examines whether a construct (along with its indicators) is empirically separate from other, theoretically unrelated constructs. Content validity, a subset of construct validity, refers to whether a measure accurately captures the intended construct, as opposed to representing an alternative domain (Johnston et al., 2014). To evaluate discriminant validity, we employed two tests: the heterotrait–monotrait ratio (HTMT) and the Fornell–Larcker criterion.

According to the Fornell–Larcker criterion, the square root of a construct’s AVE should be greater than its correlations with all other latent constructs, suggesting that the construct shares more variance with its own indicators than with those of other constructs, thus confirming discriminant validity (Al-Mekhlafi et al., 2023; Al-Mekhlafi et al., 2021).

As shown in Table 3, the boldfaced diagonal entries denote the square root of the AVE for each variable, and the off-diagonal cells report inter-construct correlations. Collectively, these results indicate adequate discriminant validity under the Fornell–Larcker criterion.

**TABLE 3:** Fornell–Larcker criterion.

Variable	ES	OCA	OCB	OCC	PCV	TI
ES	**0.767**	**0.807**	**0.830**	**0.809**	**0.820**	**0.845**
OCA	0.231					
OCB	0.222	0.368				
OCC	0.26	0.326	0.407			
PCV	0.326	0.471	0.491	0.529		
TI	0.462	0.526	0.359	0.496	0.488	

Bold values on the diagonal represent the square root of the average variance extracted ($\sqrt{\text{AVE}}$) for each construct.

HTMT is a well-established method for evaluating discriminant validity. The HTMT correlation test is often regarded as providing a superior assessment of discriminant validity. To ascertain discriminant validity, we adopted a threshold of 0.90, following prior research (Henseler et al., 2015). In our research model, the HTMT values ranged from 0.245 to 0.64, indicating that discriminant validity was satisfied.

#### 4.3.3 Collinearity diagnostics

Before analyzing the path coefficients, we examined multicollinearity. The variance inflation factor (VIF) is used in PLS to assess multicollinearity, with values below 5 indicating no concerns (Kock, 2015). In this study, the highest VIF value observed was 2.148, indicating that multicollinearity was not a problem.

### 4.4 Structural model results

The structural model was evaluated using coefficients of determination (*R*^2^), effect sizes (*f*^2^), and bootstrapped path coefficients. *R*^2^ values indicate the proportion of variance in endogenous constructs associated with their predictors.

As reported in Table 4, PCV was associated with 22.2% of the variance in OCA (adjusted *R^2^* = 0.221), 24.2% in OCB (adjusted *R^2^* = 0.240), and 28.0% in OCC (adjusted *R^2^* = 0.279). For TI, the model accounted for 50.3% of the variance (adjusted *R^2^* = 0.497), indicating substantial explanatory power. Effect sizes (*f*^2^) ranged from negligible (OCB→TI: *f^2^* = 0.003) to large (PCV→OCC: *f^2^* = 0.389), with all remaining paths exceeding the small-effect threshold.

**TABLE 4:** Model assessment results.

Variable	*R* ^2^	*R*^2^ adjusted	*f* ^2^
OCA	0.222	0.221	PCV→OCA: 0.286
OCB	0.242	0.240	PCV→OCB: 0.318
OCC	0.280	0.279	PCV→OCC: 0.389
TI	0.503	0.497	PCV→TI: 0.029
			OCA→TI: 0.156
			OCB→TI: 0.003
			OCC→TI: 0.099
			ES→TI: 0.156

**FIGURE 2:**
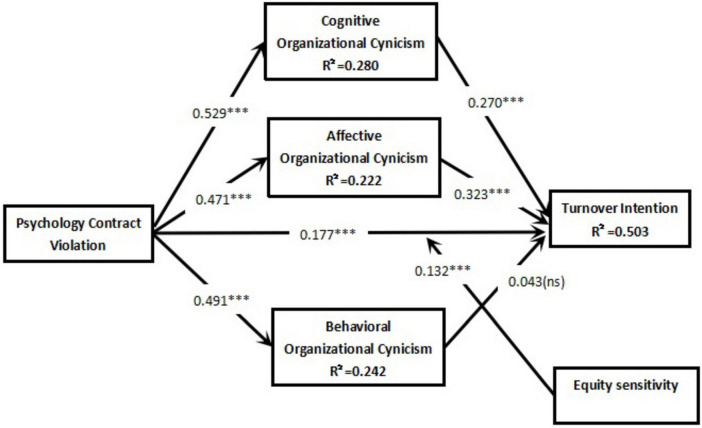
The result of the research model. ^ns^*p* ≥ 0.05, *p < 0.05, **p < 0.01, ***p < 0.001.

Bootstrapped path estimates (Figure 2 and Table 5) indicated that PCV was positively associated with OCC (β = 0.529, *p* < 0.001), OCA (β = 0.471, *p* < 0.001), and OCB (β = 0.491, *p* < 0.001), as well as with TI (β = 0.177, *p* < 0.001). In addition, OCC (β = 0.270, *p* < 0.001) and OCA (β = 0.323, *p* < 0.001) were positively associated with TI, whereas the OCB→TI path was not statistically significant (β = 0.043, *p* = 0.402).

**TABLE 5:** Direct effect summary.

Influence	β	SD	*T* statistics	*P*-values
PCV→OCC	0.529	0.056	9.443	< 0.001
PCV→OCA	0.471	0.054	8.775	< 0.001
PCV→OCB	0.491	0.064	7.660	< 0.001
PCV→TI	0.177	0.048	3.703	< 0.001
OCC→TI	0.270	0.044	6.185	< 0.001
OCA→TI	0.323	0.039	8.323	< 0.001
OCB→TI	0.043	0.051	0.838	0.402
ES × PCV→TI	0.132	0.039	3.409	0.001

### 4.5 Mediation analysis

We examined mediation effects using non-parametric bootstrapping with 5,000 resamples. An indirect effect was considered significant when the 95% bootstrap confidence interval excluded zero (Hayes, 2009).

As shown in Table 6, the indirect effects of PCV on TI via OCC (β = 0.143) and via OCA (β = 0.152) were statistically significant, whereas the indirect effect via OCB was not (β = 0.021). Following Zhao et al. (2007), the mediation patterns for OCC and OCA were classified as complementary (partial) mediation, given that both indirect and direct effects were significant and in the same direction. The non-significant indirect effect via OCB indicates that OCB does not function as a significant mediator in the sample-average model.

**TABLE 6:** Analysis of mediation.

Influence	β	SD	*T* statistics	*P*-values	2.5%	97.5%
PCV→OCC→TI	0.143	0.027	5.316	< 0.001	0.093	0.198
PCV→OCB→TI	0.021	0.025	0.848	0.397	–0.028	0.069
PCV→OCA→TI	0.152	0.024	6.380	< 0.001	0.106	0.200

### 4.6 Moderation and supplementary analyses

**FIGURE 3:**
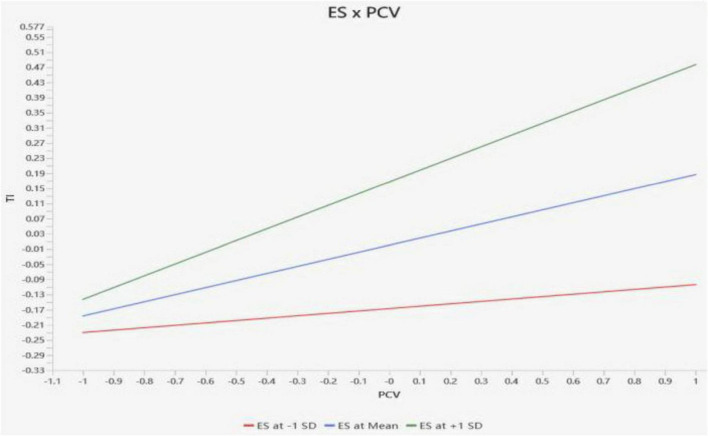
Simple slope graph.

Moderation effects were assessed by introducing interaction terms into the structural model. Moderation was considered present when the interaction coefficient reached statistical significance at *p* < 0.05. The results are reported in Table 7. Figure 3 presents a simple slope graph, illustrating the moderating effect of ES on the relationship between PCV and TI. As shown, higher levels of ES lead to a steeper increase in TI as PCV becomes more pronounced.

**TABLE 7:** Analysis of moderation.

Influence	β	SD	T statistics	*P*-values
ES × PCV→TI	0.132	0.039	3.409	0.001

As shown in Table 7, the interaction between ES and PCV (ES × PCV) was positively and significantly associated with TI (β = 0.132, *p* = 0.001), indicating that ES strengthens the positive relationship between PCV and TI. Simple slope analyses further illustrated that the PCV→TI relationship was steeper among teachers with higher levels of ES.

To further examine the non-significant mediating role of OCB, two supplementary stage-moderation analyses were conducted. First, the interaction term ES × OCB was added to predict TI, testing whether ES moderates the OCB→TI relationship. Second, the interaction term ES × PCV was added to predict OCB, testing whether ES moderates the PCV→OCB relationship. All interaction terms were mean-centered and estimated using the same bootstrapping procedure as the main model. The results of these analyses are reported in Tables 8, 9.

**TABLE 8:** ES × OCB→TI path coefficients table.

Influence	β	SD	T statistics	*P*-values
PCV→OCC	0.529	0.056	9.443	0
PCV→OCA	0.471	0.054	8.775	0
PCV→OCB	0.491	0.064	7.660	0
OCC→TI	0.266	0.043	6.223	0
OCA→TI	0.333	0.039	8.505	0
OCB→TI	0.058	0.050	1.178	0.239
PCV→TI	0.141	0.052	2.741	0.006
ES x OCB→TI	0.150	0.046	3.247	0.001

**TABLE 9:** ES × PCV→OCB path coefficient table.

Influence	β	SD	T statistics	*P*-values
PCV→OCC	0.529	0.056	9.442	0
PCV→OCA	0.471	0.054	8.775	0
PCV→OCB	0.402	0.085	4.701	0
OCC→TI	0.285	0.048	5.947	0
OCA→TI	0.345	0.048	7.197	0
OCB→TI	0.041	0.052	0.788	0.431
PCV→TI	0.154	0.049	3.152	0.002
ES × PCV→OCB	–0.091	0.049	1.869	0.062

The results indicated that the ES × OCB interaction was significant (β = 0.150, *t* = 3.247, *p* = 0.001), suggesting that ES amplifies the effect of OCB on TI. However, the main effect of OCB on TI remained non-significant. In contrast, the ES × PCV interaction predicting OCB was not significant (β = -0.091, *t* = 1.869, *p* = 0.062), indicating that ES does not substantially alter the strength of the PCV→OCB relationship. Collectively, these findings suggest that the absence of a significant OCB—mediated pathway in the baseline model is more likely attributable to the conditional nature of the OCB→TI effect rather than to suppression of the PCV→OCB link.

### 4.7 Alternative (reverse) model and model comparison

Given the single-wave survey design, an alternative structural model was estimated in which the three facets of OC (OCC, OCA, and OCB) were specified as predictors of PCV, while retaining the original paths to TI and the ES × PCV interaction.

As shown in Table 10, all three cynicism facets were significantly and positively associated with PCV, indicating that cynicism may also function as an upstream correlate of perceived violation in the present sample. Importantly, the downstream pattern of results for TI remained unchanged: OCC and OCA were positively related to TI, whereas OCB was not statistically significant.

**TABLE 10:** Reverse model path coefficient table.

Influence	β	SD	T statistics	*P*-values
OCC→PCV	0.338	0.055	6.119	0
OCA→PCV	0.267	0.045	5.955	0
OCB→PCV	0.256	0.061	4.208	0
OCA→TI	0.323	0.039	8.323	0
OCB→TI	0.043	0.051	0.838	0.402
OCC→TI	0.270	0.044	6.184	0
PCV→TI	0.177	0.048	3.707	0
ES × PCV→TI	0.132	0.039	3.413	0.001

To further assess robustness, the original and reverse models were compared using both explanatory and predictive criteria (Table 11). The two models yielded identical in-sample explanatory power for TI (*R*^2^), whereas the reverse model demonstrated higher out-of-sample predictive relevance (Q^2^predict) and lower prediction errors (RMSE and MAE). These results suggest that the focal findings for TI are robust to alternative model specifications. However, given the cross-sectional design, the analyses do not permit definitive conclusions regarding temporal precedence. Accordingly, the proposed directional model (PCV→OC→TI) is interpreted as theory-driven, with the reverse-model results viewed as consistent with potential reciprocal dynamics that warrant longitudinal investigation.

**TABLE 11:** Original and reverse model comparison.

Evaluation metric	Original model	Reverse model
*R*^2^(TI)	0.503	0.503
*Q*^2^(TI)	0.327	0.457
RMSE (TI)	0.825	0.741
MAE (TI)	0.589	0.453

## 5 Discussion

This study examines the relationships between PCV, OC, ES, and TI among Chinese preschool teachers, contributing to our understanding of retention issues in early childhood education. Our findings suggest that PCV is associated with TI through the mediating roles of OCC and OCA, with ES moderating this relationship. However, OCB did not significantly mediate the PCV—TI path.

Firstly, the association between PCV and TI is consistent with previous literature, supporting the basic premise of SET (Blau, 1964; Rousseau, 1989). Research has shown that PCV erodes trust and weakens organizational commitment, which is associated with increased TI (Li et al., 2022; Azeem et al., 2020). These findings suggest that emotional depletion (e.g., OCA) and cognitive disillusionment (e.g., OCC) are more strongly associated with TI than overt resistance in emotionally demanding jobs like teaching (Çiçek et al., 2021; Ike et al., 2024).

The partial mediation by OCC and OCA aligns with previous studies that conceptualize cynicism as a psychological response to perceived injustice or breach (Johnson and O’Leary-Kelly, 2003; Bari et al., 2022). Specifically, OCC reflects distrust and perceived ethical violations, while OCA involves negative emotional reactions such as frustration and anger. These affective and cognitive responses, rather than behavioral enactments, are more strongly associated with TI in settings characterized by high emotional labor (Çiçek et al., 2021; Ike et al., 2024).

Our study builds on these findings by highlighting the cultural context of China, where collectivist values such as harmony and respect for authority may suppress overt behavioral cynicism (Wang et al., 2025). Cultural constraints can lead to the internalization of cynicism, where teachers may feel disillusioned but refrain from public displays of dissatisfaction, such as sarcasm or passive resistance. This contrasts with more individualistic cultures, where behavioral cynicism is a more overt and significant response to PCV (Çöl, 2022).

In addition, ES positively moderates the PCV—TI relationship—individuals with higher ES, being more sensitive to violation cues, react more strongly to perceived unfairness, thereby strengthening this association (Mensah et al., 2024; Zhang et al., 2010). Moreover, the moderating role of ES is particularly pronounced when procedural justice cues are weak or inconsistent, highlighting the conditional nature of ES in collectivist contexts (Sauley and Bedeian, 2000). This is consistent with studies that have identified ES as a “psychological amplifier” that strengthens the association between perceived violations and outcomes in collectivist cultures (Mensah et al., 2024).

However, the absence of a significant OCB→TI relationship requires further exploration. We conducted supplementary interaction tests to examine whether ES influences the link between OCB and TI. Results showed that while ES significantly strengthens the association between OCB and TI, OCB was not consistently a reliable mediator in the overall model. This discrepancy suggests that behavioral cynicism may be contextually conditional, more likely to emerge among individuals with high ES, but suppressed in environments that emphasize harmony and authority (Pfrombeck et al., 2020). Additionally, cultural norms around conformity and authority in China likely mitigate the manifestation of behavioral cynicism, making it a less salient predictor of TI compared to affective and cognitive cynicism.

This research contributes to the broader understanding of the PCV—TI mechanism in collectivist societies, offering new insights into how cultural values interact with psychological mechanisms to influence TI. The study suggests that while OCB may not always be a significant predictor of TI in collectivist cultures, OCC and OCA are more strongly associated with TI, particularly when teachers feel emotionally disillusioned or distrustful. As such, future research could further explore how cultural orientations and professional norms modulate cynicism responses and affect TI in different national contexts.

Consistent with this interpretation, the alternative (reverse) model indicates that cynicism facets are also positively associated with perceived violation, suggesting that repeated skeptical cognitions and negative affect may sensitize teachers to interpret organizational practices as contract-breaking. However, because both directions are plausible in single-wave data, we rely on the theory-grounded sequencing (PCV→OC→TI) for hypothesis testing and treat the reverse-model evidence as indicative of possible feedback loops rather than as a basis for re-specifying causal claims.

In summary, our findings suggest that trust erosion and emotional disillusionment, rather than overt behavioral resistance, are more strongly associated with TI when psychological contracts are perceived to be breached. The cultural context in China, with its emphasis on harmony and respect for authority, may explain why OCB does not have a significant impact on TI in this study. Further research, particularly using longitudinal or experience-sampling designs, is needed to capture the temporal dynamics of cynicism and to clarify the contextual factors that influence its expression across cultures. This would enhance our understanding of when and why ES amplifies the effects of PCV on TI and provide more robust evidence for the causal pathways proposed in this model.

## 6 Conclusion

This study tested the relationships between PCV, OC, ES, and TI among Chinese preschool teachers, providing valuable insights into the psychological mechanisms behind teacher retention in early childhood education. The results indicate that PCV significantly influences TI, with cognitive and affective cynicism acting as partial mediators. ES was found to positively moderate the relationship between PCV and TI, highlighting the role of individual differences in shaping responses to perceived contract violations. The model explains a substantial portion of variance in TI, underscoring the predictive value of integrating PCV, OC facets, and ES in understanding TI.

### 6.1 Theoretical contributions

This study provides a contextualized application of SET within Chinese preschool education, highlighting how cultural context shapes responses to PCV. While SET traditionally focuses on reciprocity disruptions leading to negative outcomes, our findings reveal that in collectivist cultures like China, employees’ emotional reactions—specifically OCC and OCA—are more significant than OCB. This suggests that, in such environments, the response to contract violations is more internalized, with employees feeling disillusioned rather than openly defiant. This is particularly important. It underscores the need to consider cultural values—such as harmony and respect for authority—which may moderate the expression of OC in collectivist cultures. Existing research in individualistic cultures has focused predominantly on overt OCB as a primary response to perceived breaches, but our study indicates that this form of cynicism is less likely to manifest publicly in collectivist societies where maintaining social harmony is paramount. This contextualized application aligns with the call for more context-specific studies that examine the role of cultural factors in shaping employees’ responses to PCV (Wang et al., 2025; Li et al., 2022). For example, Johnson and O’Leary-Kelly (2003) highlight the role of behavioral cynicism as a primary response to PCV in individualistic settings. In contrast, our findings suggest that in collectivist cultures, cognitive and affective dimensions of cynicism play a more prominent role, pointing to a cultural moderation of the PCV—TI relationship. This contextualized analysis illustrates how SET operates in collectivist settings, suggesting that the impact of reciprocity disruptions may be shaped not just by the breach itself, but also by cultural expectations on emotional expression and social harmony. As cultural values were not directly measured, this represents an application of SET to a specific cultural context rather than an empirical extension of the theory.

Moreover, the integration of ES into the model offers a novel contribution by showing that individual differences in sensitivity to inequity—rather than general fairness perceptions—amplify the effects of PCV. Employees with high ES exhibit chronic vigilance toward inequity, which intensifies their TI when contracts are breached. Employees with high ES are more sensitive to perceived breaches, which directly impacts TI. This finding demonstrates the utility of SET in contexts where ES operates as a key moderating factor, especially in collectivist societies, where this individual difference in sensitivity to inequity may amplify outcomes differently than in individualistic settings. In particular, our findings suggest that ES may act as a “psychological amplifier” in collectivist cultures, where this heightened sensitivity to inequity can significantly magnify the negative effects of perceived violations (Mensah et al., 2024). Incorporating ES into the model provides a more nuanced understanding of how individual differences in ES shape responses to PCV, highlighting the complexity of the relationship between perceived contract breaches and TI. This contribution builds on existing research by Sauley and Bedeian (2000), who discuss the role of ES in shaping employee responses to organizational justice. However, our study is the first to apply this concept specifically to the PCV context in a collectivist society, offering a deeper understanding of how fairness perceptions can magnify the impact of a breach in such settings. Future research could explore whether fairness-oriented organizational practices could buffer the amplified effects of PCV on high-ES employees in collectivist cultures.

### 6.2 Practical implications

The findings have important implications for organizational practice, particularly in educational settings. The study underscores the need for school administrators and policymakers to address emotional disillusionment and distrust—key drivers of TI—through proactive strategies. Given that OCC and OCA emerged as significant partial mediators of the PCV—TI relationship, whereas OCB did not, administrators should prioritize interventions targeting cognitive distrust and emotional exhaustion—such as transparent communication and emotional support—rather than merely addressing overt behavioral symptoms. Within this cultural context, interventions in China should emphasize collective values, such as fostering a sense of belonging and respect for authority, to effectively mitigate emotional disillusionment. Moreover, as ES significantly moderated the relationship between PCV and TI, school administrators should screen for ES levels and calibrate management practices to accommodate individual differences in tolerance for inequity. For high-ES teachers, equity-explicit interventions—such as transparent justification for workload distributions and advance notice of policy changes—directly address their lower threshold for detecting contract breaches, thereby attenuating turnover risk. For instance, Sischka et al. (2021) recommend enhancing interpersonal communication to address perceived fairness. Our findings build on this by suggesting that fairness-oriented practices are especially crucial for equity-sensitive employees, who are particularly responsive to perceived breaches. School administrators should implement targeted interventions aimed at creating fairness through transparent performance evaluations and clear communication of role expectations, specifically for teachers with higher ES.

The finding that OCB showed no significant mediating effect, combined with the observation that PCV influenced TI primarily through cognitive and affective rather than behavioral channels, suggests that interventions relying solely on monetary incentives or contractual adjustments—which primarily address behavioral compliance—may yield limited returns. Instead, our findings indicate that resources should be directed toward mitigating the OCC and OCA that served as the primary transmission mechanisms in our model. Fostering a culture of fairness and trust within educational institutions will be critical in retaining teachers. This approach should emphasize the importance of addressing emotional needs and fairness perceptions in teacher retention strategies. Moreover, as our findings suggest that ES amplifies the effects of PCV on TI, school administrators should consider implementing fairness-oriented practices that reduce perceived inequities, especially for teachers who exhibit higher sensitivity to fairness. For example, Pfrombeck et al. (2020) emphasize leader-member exchange quality as a critical factor in moderating cynicism outcomes. Our study extends this by suggesting that improving the overall fairness of organizational practices, particularly in relation to compensation and workload distribution, could reduce TI among teachers in collectivist contexts.

Operationally, schools should monitor early signals of cognitive distrust and emotional disillusionment—the specific channels found to transmit the effects of PCV to TI—rather than focusing primarily on overt behavioral indicators such as sarcasm or passive resistance, which showed no significant association with TI in this study. Tracking these cognitive-affective markers allows administrators to deploy targeted communication before TI escalates. Schools can adopt a “monitor—respond” workflow: track early signs of OCC and OCA, identify units where disillusionment rises, and deploy targeted interventions to prevent escalation.

### 6.3 Limitations and future research

First, although we conducted an alternative (reverse) model and predictive comparisons as robustness checks, the cross-sectional self-report design cannot establish temporal precedence; future research should employ longitudinal or multi-source designs to test reciprocal dynamics between perceived violation and cynicism. The cross-sectional, self-report design restricts causal inference and introduces lingering common-method bias, even with procedural safeguards. This limitation hinders the ability to make causal claims, and future studies should utilize longitudinal, multi-source designs to more effectively establish causal relationships and mitigate biases inherent in self-report data.

Although multi-provincial, the sample may not generalize to rural, international-program, or other cultural contexts. In addition, we did not directly measure individual-level cultural values (e.g., collectivism/individualism, power distance, or harmony orientation). Therefore, our cultural explanation is offered as a context-grounded boundary explanation rather than an empirical test of cultural moderation. Future research could incorporate validated cultural value measures and/or cross-cultural samples to examine whether these values systematically condition the PCV→OC facets and OC facets→TI pathways. Non-probability sampling restricts the generalizability of the findings. To improve representativeness across various preschool settings, future research should adopt probability sampling methods.

Only ES was tested as a moderator; incorporating justice perceptions, leader–member exchange, resilience, or collectivist orientation may reveal additional contingencies. Future studies may investigate additional moderators, such as organizational justice, to further enrich our understanding of the dynamics between PCV and TI.

OCB may be phase- and norm-dependent; thus, its effects may be delayed or context-bound and better captured with panel or experience-sampling designs. The supplementary ES—OCB interaction tests were conducted as robustness-oriented, *post hoc* probes to clarify the non-significant OCB mediation. Because overt behavioral enactment is susceptible to normative constraints and self-presentation, single-source self-reports may restrict variance in OCB and attenuate its links with TI. Future studies should replicate the ES-contingent behavioral chain using longitudinal and/or multi-source indicators and test conditional indirect effects over time.

In conclusion, this study enriches our understanding of how PCV, OC, and ES interact to influence TI among preschool teachers in China. By offering a deeper understanding of these mechanisms, the study provides a foundation for developing more effective strategies to address teacher retention challenges in China and other collectivist cultures.

## Data Availability

The original contributions presented in this study are included in this article/Supplementary material, further inquiries can be directed to the corresponding author.
