# Supplementary figures and images for: Understanding turnover intention among preschool teachers in China: the role of psychological contract violation, organizational cynicism, and equity sensitivity
Source: Front Psychol. 2026 Mar 11;17:1734017. doi: 10.3389/fpsyg.2026.1734017 (PMC13013043; doi:10.3389/fpsyg.2026.1734017)

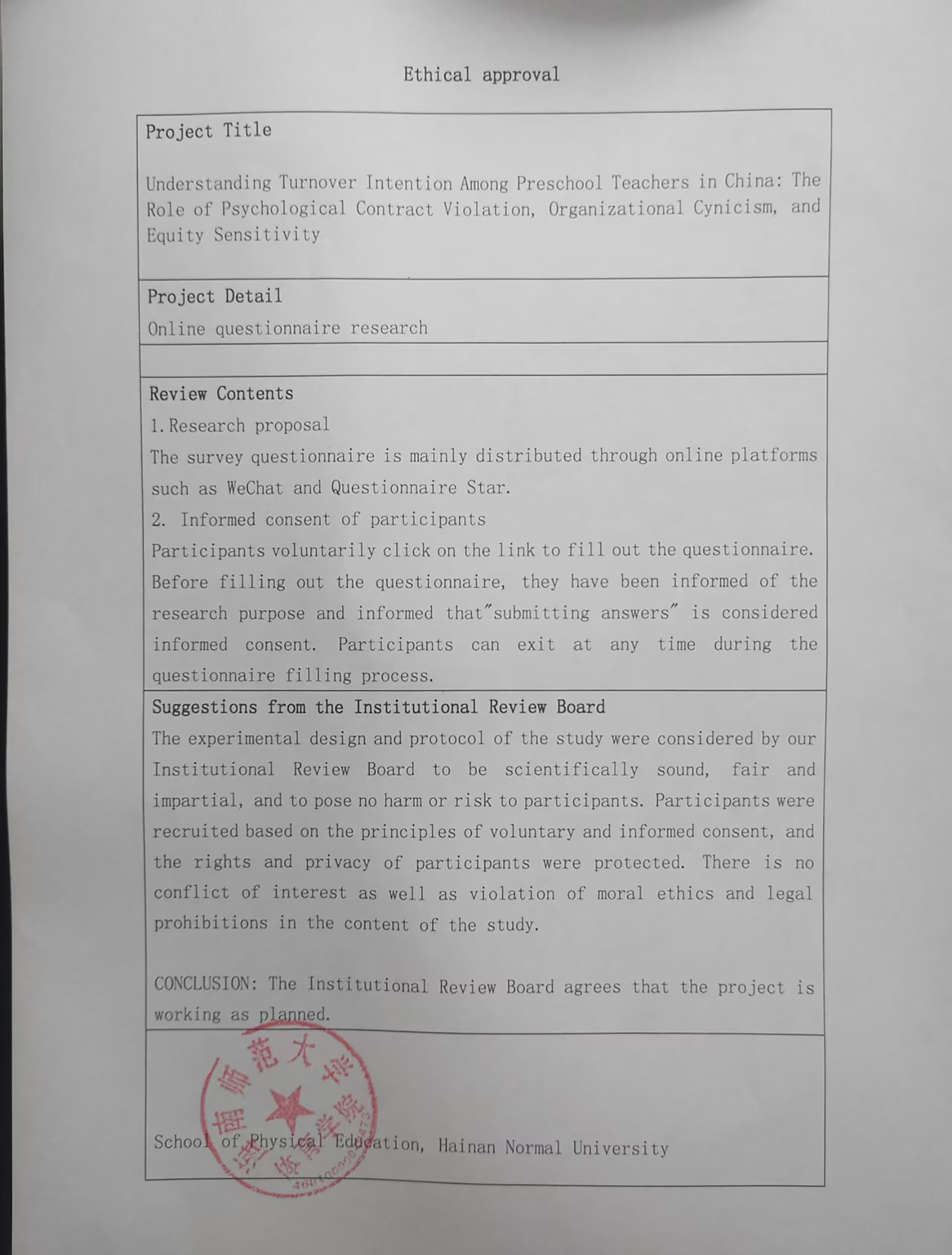

Supplement: Supplementary file 2 [file Image_1.JPEG]
